# Peritoneal dissemination of ascending colon cancer demonstrating relapse-free survival for 40 months with panitumumab monotherapy: A case report

**DOI:** 10.1016/j.ijscr.2019.05.001

**Published:** 2019-05-09

**Authors:** Katsuji Tokuhara, Nobuyuki Yamamoto, Hidehiko Hishikawa, Kazuhiko Yoshioka

**Affiliations:** Department of Surgery, Kansai Medical University, 10-15 Fumizono, Moriguchi, Osaka 570-8507, Japan

**Keywords:** Metastatic colorectal cancer, Peritoneal dissemination, Panitumumab, Anti-EGFR antibody

## Abstract

Introduction: The prognosis of metastatic colorectal cancer (mCRC) patients receiving multiple cytotoxic agents and targeted therapies (CATT) has improved, but a complete cure by CATT is still very rare.

Presentation of case: We report the successful treatment of ascending colon cancer complicated by peritoneal disseminations (PDs) with panitumumab (Pmab) plus mFOLFOX6 therapy. A 67-year-old male patient was diagnosed with clinical stage IV cancer of the ascending colon with PDs, and underwent ileostomy. Eighteen courses of Pmab plus mFOLFOX6 caused remarkable tumor shrinkage and the disappearance of PDs on ECT. Laparotomy revealed tumor shrinkage and scarring at the PD sites. We performed right hemicolectomy, subtotal omentectomy, and ileostomy closure as curability B surgery. Seven months later, new PDs were detected by ECT so we resumed Pmab plus mFOLFOX6 therapy. After nine courses of treatment, the target lesion had completely disappeared. After a total of 20 courses, we changed to Pmab monotherapy as maintenance therapy because there was no recurrence. Forty months after the initiation of Pmab monotherapy, there has been no oncologic progression.

Discussion: Pmab plus mFOLFOX6 treatment resulted in a complete response for PDs, which is extremely rare for CATT.

Conclusion: We consider that Pmab therapy should be introduced for the treatment of mCRC complicated by PDs.

## Highlights

• We report a colon cancer patient complicated by peritoneal dissemination.
• The patient was completely cured with Pmab plus chemotherapy.
• Pmab therapy should be considered as a treatment option for peritoneal dissemination.

## 1 Introduction

Multiple cytotoxic agents and targeted therapies (CATT) have been developed for the treatment of metastatic colorectal cancer (mCRC). As a result, patient prognosis has been improving, and the median survival time is now 20–30 months [1]. However, achieving a cure for mCRC patients using CATT is still very rare. Here, we report a case of ascending colon cancer complicated by peritoneal dissemination that was completely cured by panitumumab (Pmab) plus multiple cytotoxic agent therapy (CAT). This case report is compliant with SCARE Guidelines [2].

## 2 Presentation of case

A 67-year-old man was referred to our hospital to determine the cause of his diarrhea and nausea. Colonoscopy revealed a type II tumor located in the ascending colon. Histopathological findings of the biopsy specimen of the ascending colon tumor revealed well–moderately differentiated tubular adenocarcinoma. The K-ras status was wild-type and it was epidermal growth factor receptor (EGFR)-positive.

**Fig. 1:**
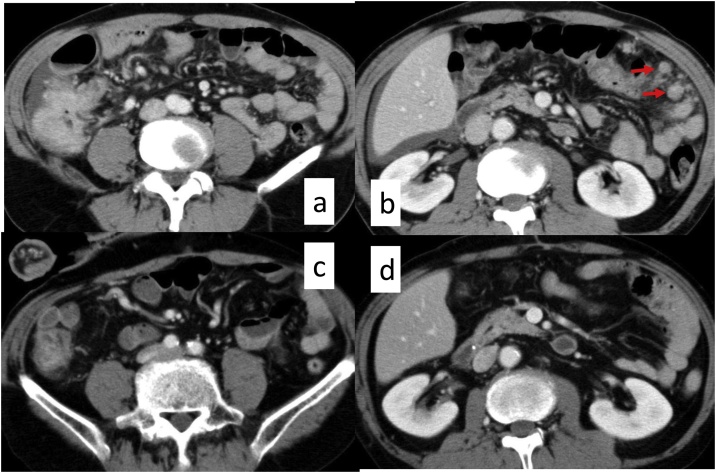
Thoracic–abdominal enhanced computed tomography (ECT). A, B. ECT showing the wall thickness of the ascending colon, ascites in the pelvic cavity and right upper abdomen, and suspected peritoneal disseminations (PDs) (arrow) in the left upper abdomen. C, D. After 18 courses of chemotherapy, the volume of the ascending colon cancer had shrunk to around 50% and peritoneal disseminations and ascites had disappeared.

**Fig. 2:**
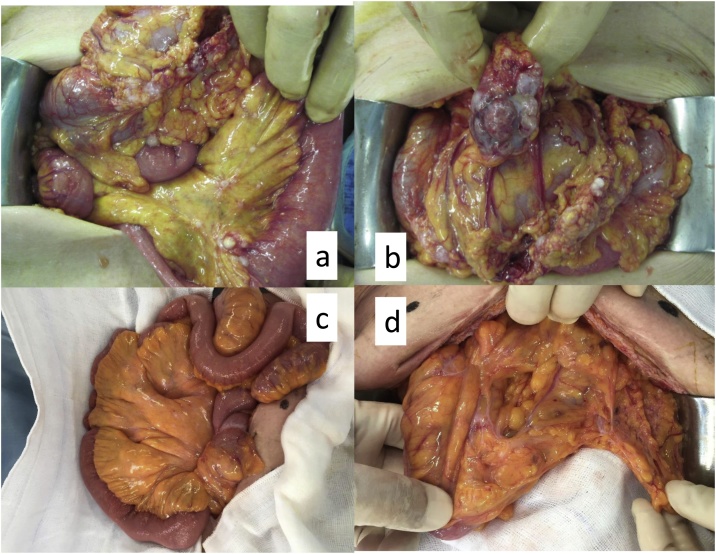
Intraoperative findings. A, B. Extensive PDs in the peritoneal cavity including the greater omentum. C, D. After 18 courses of Pmab plus mFOLFOX6, the PD sites appeared as white scarring and ascites cytology was negative for adenocarcinoma.

Thoracic and abdominal enhanced computed tomography (ECT) showed the wall thickness of the ascending colon, ascites in the pelvic cavity and right upper abdomen, and suspected peritoneal disseminations (PDs) in the left upper abdomen (Fig. 1a, b). Blood examination revealed no abnormalities, except for the level of carcinoembryonic antigen (CEA) (8.6 ng/ml). We therefore diagnosed clinical stage IV cancer [cT4a, N2a, M1a(P3)] of the ascending colon [3]. The patient had an obstructed bowel because of the tumor, so we performed surgery to remove it. Intraoperatively, we observed extensive PDs in the peritoneal cavity including the greater omentum (Fig. 2a, b). We therefore abandoned our planned curative resection because we considered that an intestine–colon bypass would have a high risk of anastomotic leakage because of the PDs; instead, we performed loop ileostomy. Ascites cytology was positive for adenocarcinoma.

Two weeks after the ileostomy, we administered systemic chemotherapy consisting of Pmab plus mFOLFOX6 (panitumumab, 6 mg/kg; oxalliplatin, 85 mg/m^2^; bolus injection of 5FU, 400 mg/m^2^; continuous injection of 5FU, 2400 mg/m^2^/48 h) every 2 weeks. After 18 courses of this chemotherapy, abdominal ECT showed that the tumor volume had shrunk to around 50% and PDs and ascites had disappeared (Fig. 1c, d). The level of CEA in the blood also decreased from 28.3–4.3 ng/ml.

At this time, the patient hoped to undergo ileostomy closure surgery and we considered laparotomy useful to evaluate the anti-tumor effect of Pmab plus CAT. Intraoperatively, the PDs were visible as white scarring and ascites cytology was negative for adenocarcinoma (Fig. 2c, d). The tumor had shrunk remarkably and there was no invasion of the retroperitoneum. We therefore considered that curability B resection was suitable, so the patient underwent right hemicolectomy, subtotal omentectomy, and ileostomy closure surgery.

**Fig. 3:**
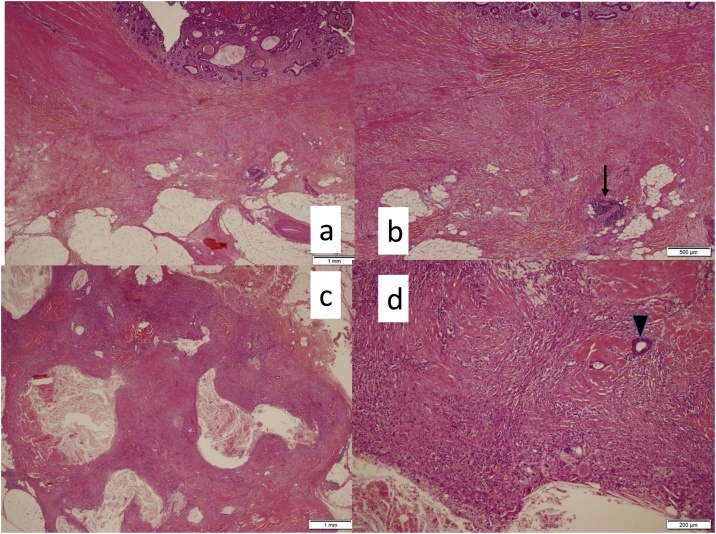
Histopathological findings. A, B. Hematoxylin and eosin (HE) staining of ascending colon cancer showing extensive fibrosis from the submucosa to the subserosal tissue with ductal adenocarcinoma duct (arrow) in the muscularis propria. C, D. HE staining of the omentum revealing almost burned-out lesions with ductal adenocarcinoma (arrowhead).

Histopathological findings based on hematoxylin and eosin (HE) staining of the tumor revealed extensive fibrosis from the submucosa to subserosal tissue with ductal adenocarcinoma duct in the muscularis propria. Metastasis was confirmed in three out of 14 regional lymph nodes, although two of the three metastasic lymph nodes were burned-out by Pmab plus CAT treatment. HE staining of the omentum revealed almost burned-out lesions with some duct adenocarcinoma (Fig. 3a–d). The histopathological anti-tumor effect of the chemotherapy was grade 1b, and we diagnosed ypStage IV cancer [ypT2, N1b, M1a(H0P1PUL0)] of the ascending colon [3].

**Fig. 4:**
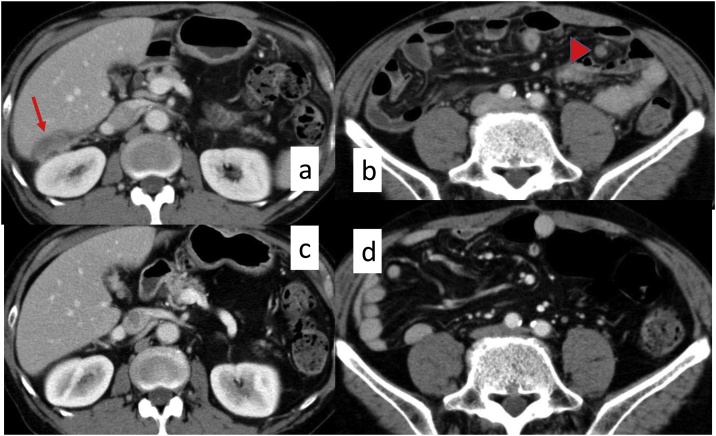
ECT. A, B. New PDs detected by follow-up abdominal ECT 7 months after surgery. C, D. The target lesion had completely disappeared after nine courses of Pmab plus mFOLFOX6 for new PDs.

Although we had undertaken clinical follow-up after the operation without through patient choice and because there were no target lesions, new PDs were detected by follow-up abdominal ECT (Fig. 4a, b) 7 months after surgery. Therefore we resumed Pmab plus mFOLFOX6 (Pmab, 6 mg/kg; oxalliplatin, 85 mg/m^2^; bolus injection of 5FU, 400 mg/m^2^; continuous injection of 5FU, 2400 mg/m^2^/48 h) for new PDs. After nine courses of this therapy, the target lesion had completely disappeared on ECT (Fig. 4c, d), so we evaluated this therapy as a complete response (CR). After 20 courses of this therapy, we changed the treatment regimen from Pmab plus mFOLFOX6 to Pmab monotherapy as maintenance therapy because no recurrent lesions had appeared. We have been continuing Pmab monotherapy because it controls PDs and there have been no marked adverse events. We have also continued strict follow-up by ECT every 2–3 months and there has been no oncologic progression 40 months after the initiation of Pmab monotherapy.

## 3 Discussion

The incidence of CRC has increased gradually in worldwide. In Japan in 2017, CRC was ranked as the second and third most common cancer among women and men, respectively [4]. It is also the second leading cause of cancer death in Japan. PDs occur in 4.5% of Japanese mCRC patients, and this rate is second only to that of liver metastasis at 10.9% [5]. The prognosis of CRC patients with PDs was shown to be poor compared with other organ metastases such as those of the liver and lung, with a 1-year survival rate of 33% [6].

Standard treatment for mCRC has been CAT with biologic drugs such as anti-vascular endothelial growth factor (VEGF) or anti-EGFR antibodies. Pmab is one such anti-EGFR antibody, and Pmab plus CAT treatment previously resulted in a better prognosis for mCRC than CAT without target therapies. Indeed, anti-EGFR antibodies plus CAT tend to cause early tumor shrinkage which improves prognosis [7]. The regular dose of Pmab is 6 mg/kg, which is introduced in combination with CAT such as oxaliplatin or irinotecan plus 5FU every 2 weeks; Pmab monotherapy is also effective for mCRC. Pan-Asian adapted European Society for Medical Oncology and National Comprehensive Cancer Network (NCCN) guidelines recommend Pmab plus CAT from first line to late-line therapy for mCRC, especially left-sided CRC [8,9].

Although CATT has been effective for prolonging life, very few patients have achieved a CR for mCRC [10,11]. Qi et al. reported an overall incidence of CR in patients treated with CATT of 2.4% compared with 1.3% for CAT [12]. In our case, Pmab plus CAT led to a CR for P3 from ascending colon cancer, and Pmab monotherapy maintained a CR. Only one case report has previously described a CR following Pmab treatment, but the recurrence status was unknown [13]. In this report, the target lesion was lung metastasis and the primary focus was left-sided adenocarcinoma of the colon. Pmab plus FOLFIRI as second-line chemotherapy induced CR, after which FOLFIRI was discontinued for toxicity, Pmab monotherapy was continued, and the patient maintained a CR.

NCCN guidelines recommend anti-EGFR antibody treatment as first line therapy for left-sided colon cancer but not for metastatic right-sided colon cancer [9]. Instead, anti-VEGF receptors such as bevacizumab plus CAT are recommended as first line therapy. In our case, although the primary focus of our patient was the right-sided colon, we introduced Pmab plus CAT which led to a CR for PDs (P3). Moreover, Pmab monotherapy as maintenance retained the CR. Therefore, we suggest that even if the primary focus is the right-sided colon, an anti-EGFR antibody plus CAT should be introduced for mCRC patients who require quick and marked reduction of metastatic lesions that cause concomitant symptoms such as ileus or pain.

The prognosis of CRC patients complicated by PDs is poor compared with other organ metastases such as those of the liver and lung. Moreover, PDs diminish the quality of life by ascites retention, malnutrition, and intestinal obstruction. Bevacizumab plus CAT was reported to be superior to cetuximab plus CAT for patients complicated by PDs as measured by progression-free survival (PFS) and overall survival (OS). Bevacizumab-based triplet chemotherapy was also superior to cetuximab-based triplet chemotherapy as measured by PFS (9.6 vs 6.1 months, respectively) and OS (26.3 vs 12.7 months, respectively), but not for patients without PDs (PFS, 10.6 vs 9.1 months, respectively; OS, 27.9 vs 30.7 months, respectively; p < 0.05) [14]. Based on these findings, our patient was a rare and important case as an example of CRC with PDs that achieved a CR following Pmab plus CAT treatment. Therefore, we propose that anti-EGFR antibody plus CAT should be considered as a treatment option for PDs.

## 4 Conclusions

We report a case of metastatic ascending colon cancer complicated by PDs that was successfully treated with the anti-EGFR antibody Pmab plus CAT. We consider that Pmab plus CAT could be introduced for mCRC complicated by PD.

## Conflicts of interest

The authors declare that they have no conflict of interests.

## Funding

None.

## Ethical approval

This paper was not a research study, so ethical approval not required.

## Consent

I got written informed consent from the patient for publication of this case report.

## Author contribution

KT made a substantial contribution to study conception, conducted a literature search, and drafted the manuscript. KT, NY, HH, and TY contributed to the acquisition of data. KT performed the operation. KT, NY, HH, TY, and KY reviewed the manuscript and gave final approval for publication. All authors read and approved the final manuscript.

## Registration of research studies

This literature is case report, not research study.

## Guarantor

The Guarantors of this manuscript are Katsuji Tokuhara and Prof. Kazuhiko Yoshioka.

## Provenance and peer review

Not commissioned, externally peer-reviewed.
